# A Pervaporation Study of Ammonia Solutions Using Molecular Sieve Silica Membranes

**DOI:** 10.3390/membranes4010040

**Published:** 2014-02-17

**Authors:** Xing Yang, Thomas Fraser, Darli Myat, Simon Smart, Jianhua Zhang, João C. Diniz da Costa, Audra Liubinas, Mikel Duke

**Affiliations:** 1 Institute for Sustainability and Innovation, College of Engineering and Science, Victoria University, P.O. Box 14428, Melbourne, Victoria 8001, Australia; E-Mails: xing.yang@vu.edu.au (X.Y.); t.fraser3@student.unimelb.edu.au (T.F.); darli.myat@vu.edu.au (D.M.); jianhua.zhang@vu.edu.au (J.Z.); 2 FIMLab—Films and Inorganic Membrane Laboratory, School of Chemical Engineering, The University of Queensland, Brisbane, QLD 4072, Australia; E-Mails: s.smart@uq.edu.au (S.S.); j.dacosta@eng.uq.edu.au (J.C.D.C.); 3 City West Water, Melbourne, VIC 3020, Australia; E-Mail: aliubinas@citywestwater.com.au

**Keywords:** molecular sieve silica membrane, pervaporation, vacuum membrane distillation, ammonia removal, competitive adsorption

## Abstract

An innovative concept is proposed to recover ammonia from industrial wastewater using a molecular sieve silica membrane in pervaporation (PV), benchmarked against vacuum membrane distillation (VMD). Cobalt and iron doped molecular sieve silica-based ceramic membranes were evaluated based on the ammonia concentration factor downstream and long-term performance. A modified low-temperature membrane evaluation system was utilized, featuring the ability to capture and measure ammonia in the permeate. It was found that the silica membrane with confirmed molecular sieving features had higher water selectivity over ammonia. This was due to a size selectivity mechanism that favoured water, but blocked ammonia. However, a cobalt doped silica membrane previously treated with high temperature water solutions demonstrated extraordinary preference towards ammonia by achieving up to a 50,000 mg/L ammonia concentration (a reusable concentration level) measured in the permeate when fed with 800 mg/L of ammonia solution. This exceeded the concentration factor expected by the benchmark VMD process by four-fold, suspected to be due to the competitive adsorption of ammonia over water into the silica structure with pores now large enough to accommodate ammonia. However, this membrane showed a gradual decline in selectivity, suspected to be due to the degradation of the silica material/pore structure after several hours of operation.

## 1. Introduction

Ammonia is a valuable chemical, mainly produced for agricultural purposes and chemical production [1]. Conventionally, it is synthesised through the energy intensive Haber Bosch process, which consumes up to 3% of the world’s energy usage and has a price of US$ 460–745 per tonne. At the same time, ammonia (or nitrogen compounds in other forms derived originally from ammonia) is considered as a waste by-product in industrial and municipal wastewater. The loss of ammonia to sewers and other sources leads to various problems: as a highly soluble compound in water, its accumulation in water leads to eutrophication and depletion of oxygen and, thus, harms aquatic life [2,3,4]; and the volatile form of ammonia (at its native higher pH) can be trapped in sewer headspaces, presenting a safety issue to workers, leading to costly neutralisation. Therefore, removing it prior to discharge to sewers or the environment would be preferable to nitrogen removal at wastewater treatment plants, which is an energy intensive process. An environmentally friendly alternative is to find solutions to isolate ammonia from wastewater, potentially capturing it for reuse as a valuable chemical.

Conventional techniques have been widely used to remove ammonia from wastewater effluents, such as lime treatment/air stripping, break-point chlorination, ion-exchange and biological nitrification-denitrification processes [2,5,6,7,8]. However, the applicability of these ammonia management techniques is subject to several factors: the ammonia concentration and pH levels of the feed, operational complexity, production outputs and energy costs. The major challenges to be addressed for most existing ammonia management processes for safe disposal include low single-stage removal efficiency, a huge footprint, massive chemical addition, ecologically unfriendly wastes/by products and intensive consumption of thermal energy. Instead, a direct capture technique is proposed to avoid the above issues by recovering ammonia as a resource with simple operating procedures, a small footprint and high single-stage efficiency, as well as low energy requirements.

Since the 1980s, membrane-based gas adsorption/stripping processes have been widely employed to remove ammonia from industrial wastewater; however, the low efficiency with respect to energy consumption and costly regeneration processes have limited its applications for treating feeds with a wide range of concentrations [9]. In recent decades, membrane distillation (MD), a thermally-driven process involving heat and mass transfer across a hydrophobic membrane, has received much attention, due to its versatility in various industrial applications treating difficult brine effluents. Advantages, such as the mild operating conditions of low pressure and temperature, as well as the capability of utilizing low-grade heat, such as power plant waste heat, solar and geothermal energy [10,11,12,13], have made MD an energy competitive desalination technology [13,14,15,16,17]. More recently, MD has been used to remove volatile compounds from various wastewaters with no chemical addition [18], where ammonia is of particular interest [19,20]. Compared to three other MD configurations (*i.e*., direct contact MD (DCMD), sweeping gas MD (SGMD), air gap MD (AGMD) [21,22]), vacuum MD (VMD) was considered the most effective for removing volatile compounds from aqueous solutions [19,23]. It was reported that more than 90% ammonia removal could be achieved in a single pass system using polytetrafluoroethylene (PTFE) hydrophobic membranes in VMD, with a separation factor of ammonia of up to eight times [19]. Nevertheless, given the difficulty of separating the ammonia-water system above its inherent thermodynamic limits, the existing MD processes are still strongly inhibited from achieving a high ammonia downstream concentration for direct reuse in industry. Thus far, no attempts have been made to concentrate ammonia using MD. Therefore, a more effective process with highly selective membranes is desired for recovering ammonia from aqueous solutions.

Molecular sieve silica-based ceramic membranes have been considered to separate H_2_ (a similar kinetic molecular diameter to H_2_O, 0.289 nm) from H_2_/NH_3_ gas mixtures, due to the inherent size selectivity. However, a previous study [24] has also reported poorer H_2_/NH_3_ selectivity for a silica membrane with slightly larger pore sizes of about 0.35 nm. In this case, the membrane exhibited higher selectivity toward NH_3_ (molecular size ~0.326 nm) at low temperatures (<100 °C), because of stronger competitive adsorption between silica and NH_3_ over H_2_. Other studies have reported a novel two-step system [25] using a cellulose membrane pervaporation (PV) system to separate water and ammonia from urine, followed by a silica adsorption column to completely remove ammonia and recover pure water. In recent years, the application of molecular sieve silica membranes has been broadened to the water treatment industry [26,27]. In 2007, the silica-based ceramic membrane was first introduced to pervaporative desalination [26], where selective diffusion of water vapour through the silica matrix, at the exclusion of salts, was observed. Given the molecular size difference between water (vapour form) and ammonia (vapour form), as well as a stronger competitive adsorption between silica and ammonia, an effective separation of ammonia from the aqueous environment using a molecular sieve silica membrane is expected to be feasible. However, thus far, no open literature is available on this subject.

Therefore, this study attempts to explore the performance of molecular sieving silica membranes for the pervaporation of ammonia solutions. Using a hydrophobic polypropylene (PP) membrane as the benchmark, a series of molecular sieve silica-based ceramic membranes were evaluated in terms of the ammonia concentration factor and long-term membrane performance. The goal here is to capture ammonia in the permeate for reuse, so a new experiment had to be developed, which involved the capture and analysis of the permeate solution. Furthermore, the main mass transfer mechanism across the silica membrane matrix is discussed based on the combined effect of molecular scale diffusion and competitive adsorption.

## 2. Experimental

### 2.1. Membrane Materials and Surface Modification

For comparison, two inorganic silica-based ceramic membranes with different material chemistries were prepared by coating a thin silica film onto the γ-alumina substrates via the sol-gel method. It is anticipated that they will exhibit molecular sieving function [24]. Additional metal doping using cobalt and iron was applied on the two ceramic membranes, namely CoSi and FeSi, to enhance the hydrostability in treating aqueous solutions [28]. The detailed membrane preparation and metal doping procedure can be found in the literature [29]. Briefly, cobalt nitrate hexahydrate [Co(NO_3_)_2_·6H_2_O; 98%, Alfa Aesar, Heysham, UK], iron nitrate nonahydrate [Fe(NO_3_)_3_·9H_2_O; 98%, Alfa Aesar], absolute ethanol (EtOH; AR grade, Ajax, Teran Point, Australia), tetraethyl orthosilicate (TEOS, Aldrich, St. Louis, MO, USA) and hydrogen peroxide [H_2_O_2_, AR grade (30 wt % in water), Ajax, Teran Point, Australia] were used without further purification. Commercial tubular porous alumina substrates were obtained from the Energy Research Centre of the Netherlands (Petten, The Netherlands) for CoSi and from Ceramic Oxide Fabricators for FeSi. The cobalt silica sol was prepared by dissolving an appropriate amount of cobalt nitrate hexahydrate Co(NO_3_)_2_·6H_2_O in hydrogen peroxide. Ethanol (EtOH) and tetraethyl orthosilicate (TEOS) were then added to the sol to form a final molar ratio of Co:TEOS:EtOH:H_2_O_2_ = 1:4:255:5. By contrast, the iron silica sol was dissolved in EtOH, before TEOS was added drop-wise to obtain a molar ratio of Fe:TEOS:EtOH = 1:4:255. Both sols were stirred for 3 h in an ice-cooled bath to completely hydrolyse the TEOS. The membranes were coated with the appropriate sol-gel using a custom dip-coater. The sol-gel coatings used an immersion time of 1 min and immersion/withdrawal speed of 10 cm·min^−1^, with subsequent calcination in air at 630 °C for 2 h and a heating/cooling rate of 1 °C·min^−1^. A total of 6 coatings were needed for both membranes.

To explore the effect of pore size and the structure of the silica membranes, the CoSi membrane was further treated in hydrothermal conditions to achieve pore expansion by immersing into 90 °C hot water for over one week, namely “CoSi-treated”. All membranes were dried before testing.

The detailed specifications for polymeric hollow fibres and ceramic tubes were obtained through conventional characterization techniques and gas separation tests, respectively, the results of which are given in Table 1. The hydrophobic polypropylene (PP) membrane was provided by Memcor Australia. The membrane is produced for the microfiltration of water and is not manufactured or marketed specifically for the purpose of membrane distillation, but was utilised in this work for its hydrophobic property to benchmark the ammonia separation performance by VMD. To perform the VMD testing, the membranes were potted into copper housings of 0.4 m in effective length to fabricate membrane modules. The effective membrane areas of various modules are also given in Table 1.

**Table 1:** Characterization data for various membranes. PP = polymer membrane.

Membrane Type	Membrane material	Metal dopant	Pore size (μm)	Inner/outer diameter (mm)	Contact angle (°)	Surface area of tested module (m^2^)
CoSi	Silica-based Ceramic	Cobalt	He/N_2_ gas separation factor 3.0	8/10	–	0.00740
FeSi	Silica-based Ceramic	Iron	He/N_2_ gas separation factor 5.0	8/10	–	0.00175
PP	Polypropylene	–	0.2	0.25/0.5	118° ± 6°	0.00377

### 2.2. Experimental Setup

**Figure 1:**
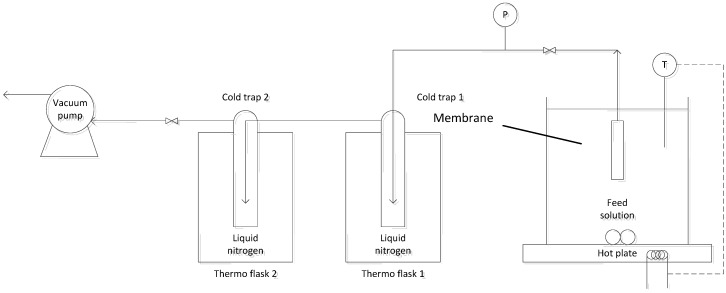
Experimental setup.

To evaluate the ammonia capture performance for various membranes, the following experiments were carried out: (i) attainable flux experiments in which the feed temperature was varied and other operating conditions remained constant; and (ii) long-term tests to observe the membrane degradation effect in which all the operating conditions are constant. Both the PV and VMD experiments were conducted using the same system. The experimental setup is shown in Figure 1, in which the hollow fibre module was submerged into the feed solution (temperature range of 25−50 °C) with a vacuum (100–150 Pa) applied on the permeate side with an oil diaphragm pump. Feed solution mixing was controlled using a stirrer bar with a speed of 760 rpm, and a complete immersion of all fibres was ensured to avoid experimental errors from atmospheric vapour permeating into the system. Two cold traps containing liquid nitrogen were placed along the vacuum line to capture the vapour generated. The product was collected and measured at 30-min intervals. The second trap simply offered protection for the oil diaphragm pump from corrosion caused by uncondensed volatiles. All samples were analysed three times and showed reproducible results.

Different feeds were prepared and tested at varied operating conditions. With a base solution of constant salinity of 500 mg/L [*i.e*., sodium chloride (NaCl) dissolved in deionized (DI) water], two synthetic feed solutions with ammonia concentrations of 50 and 1000 mg/L were prepared by dissolving ammonium hydroxide (28%–30% NH_3_, Sigma-Aldrich, St. Louis, MO, USA) into the base solution. The pH of the two feeds reached 9 and 10 at 25 °C, respectively. Furthermore, DI water was used to simply investigate the effect of feed temperature on the treated CoSi membrane, compared to the benchmark PP membrane.

To ensure all the ammonia was captured for analysis, 1.0 wt % sulphuric acid (H_2_SO_4_) solution was added to the nitrogen traps to capture the ammonia downstream (permeate side of the membrane) for product concentration analysis, when collecting the product. This is because the H_2_SO_4_ solution reacts with the frozen ammonia to form non-volatile ammonium to prevent the loss of its relatively small quantities captured in the laboratory setting:

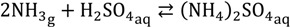
(1)

The total nitrogen concentration of the feed and permeate samples was measured using the Total Nitrogen unit (model No.: TNM-1) of the Shimadzu TOC/TN analyser, where the bound nitrogen is converted to detectable nitrogen form (*i.e*., NO_2_) in an oxygen rich combustion tube and detected by the chemiluminescence detector [2]. Prior to the test, a calibration curve was established for a testing range of 0–50 mg/L using known KNO_3_ standard solutions. Then, all permeate samples were diluted to a measurable level using DI water. With the total nitrogen concentration ([N]) obtained from the TN analyser, the ammonia concentration ([NH_3_]) can be determined using the following Equation:

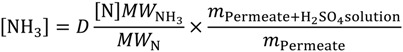
(2)

where *D* is the dilution factor using DI water, *m* dictates the total mass of the sample, in kilograms. *MW* is the molecular weight, in grams per mole. The TN technique for ammonia analysis was validated by measuring the synthetic ammonia feed solutions. The measurement error for all sample tests is within 3%.

## 3. Theory

### 3.1. Ammonia/Ammonium Dissociation Reaction

The ammonia in wastewater effluents has two forms: as free volatile ammonia and ammonium ions. The equilibrium between two forms follows the chemical reaction [30]:

NH_3_ + H_2_O ↔ HN_4_^+^ + OH^-^ (3)

A more efficient ammonia treatment process is desired to maximize the removal/separation of the volatile ammonia component. However, the amount of ammonia in free form that can be removed is highly dependent on the temperature of the aqueous solutions. For example, at a temperature of 25 °C, the equilibrium constants of the above chemical reaction to NH_4_^+^ is 3.2 × 10^4^ times higher than towards the NH_3_ [30]. The solubility of ammonia decreases significantly with increasing feed temperature and increasing pH values based on Equation (3) [19]. Therefore, high temperature and high pH would favour the capture of free ammonia from aqueous solutions. To identify the capture efficiency and the possibility of ammonia reuse, the concentration factor of ammonia, α, is defined as:

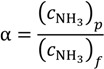
(4)
where 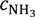 is the ammonia concentration in the aqueous solution, in milligrams per litre; symbols *p* and *f* denote the permeate and feed streams. Another term, 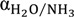 , is used to characterize the water/ammonia separation factor of a membrane as:

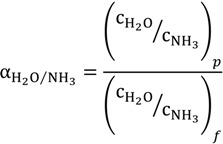
(5)

For dilute aqueous solution with low ammonia concentration, α_H_2_O/NH_3__ can be approximated to 1/α.

The separation of the ammonia-water mixture can be achieved by either conventional distillation or membrane distillation technologies. However, the separation performance is subjected to the thermodynamic constraints of the system based on the volatility (boiling point) difference of the various substances (e.g., −33.4 °C for ammonia and 100 °C for water at atmospheric pressure) [31]. Based on Henry’s law [32,33], the partial pressure of ammonia and total vapour pressure of 1000 mg/L of aqueous solution can be calculated as 104 Pa and 3270 Pa at 25 °C and 474 Pa and 9120 Pa at 45 °C, respectively. Hence, the classic thermodynamics for free surface distillation gives the aqueous solution concentration factor of NH_3_ of 30 and 59 at 25 °C and 45 °C, respectively. For instance, in the MD system where the polymeric MD membranes with hydrophobic surface properties are commonly employed, only water vapour and volatile compounds are allowed to penetrate through the membrane pores. At the mouth of the membrane pores at the aqueous feed side, a vapour-liquid interface is created between the liquid feed and vapour permeate, where the classic thermodynamic relationship applies [31]. However, due to the existence of concentration/temperature polarization effects, membrane separation outcomes (e.g., MD) are expected to be lower than the thermodynamic values.

### 3.2. Transport Mechanism through Molecular Sieve Silica Membrane in Desalination

Molecular sieve silica membranes are usually used for gas separation. due to the good fit between the pore size range and the kinetic diameters various gases, for example: He, H_2_, NH_3_ and N_2_ [24], among others. In general, size exclusion (molecular sieving) is considered as the governing diffusion mechanism. As stated in a prior study [24] on gas separation of an ammonia and hydrogen binary gas mixture, in porous silica membranes with a pore size smaller than the molecular size of ammonia (0.326 nm), the species permeation and membrane selectivity behaviours are mainly determined by size exclusion (molecular sieving). Hence, the permeation of hydrogen (molecular size of 0.289 nm) was more dominant through such a membrane. Similarly, the water molecules (vapour form) are anticipated to show a higher selectivity than ammonia, due to their much smaller size of 0.26 nm. However, the same researchers [24] have also observed that a slight increase in the pore size to 0.35 nm, which is slightly larger than the size of ammonia, did allow a more selective permeation of the ammonia over hydrogen. This is due to the combined effect of molecular scale diffusion and adsorption, which is associated with the strong interaction of ammonia with acidic silica sites within the molecular dimensioned membrane structure. Subsequently, the ammonia permeation dominates, and hydrogen molecules (or water molecules in the case we propose here) are effectively blocked.

Aiming at improving the ammonia selectivity of the current molecular sieve silica membranes, hydrothermal treatment (long exposure to a hot water environment) was reported to be effective in modifying the silica pore structure to a desired level [34]; the reason being that the migration of silica to smaller micropores has led to pore widening as the surface seeks to minimise its surface energy. A well-controlled hydrothermal treatment would induce the desired membrane pore expansion and possibly alter the permselectivity of different species. For instance, in the separation of ammonia and water vapour, as the pore widening (~0.4 nm) occurs in the hydrothermally treated silica membrane, the selectivity towards ammonia is more preferable and is strongly associated with the combined mechanism of molecular diffusion and competitive adsorption. Thus, the potential of the ceramic membranes would be expected to exceed that of conventional VMD by presenting an additional selective driving force over the thermodynamics.

Nevertheless, a further increase on the membrane pore size would instead result in a loss in separation efficiency. In this case, the adsorption of NH_3_ molecules is no longer able to block the permeation of other small molecules. Furthermore, this is associated with membrane degradation, due to overexposure to hydrothermal conditions (e.g., submerged membrane modules). Hence, the fabrication of silica membranes with high hydrothermal stability is essential. Novel methods were proposed to improve the ammonia stability of the silica membranes in the treatment process of aqueous solutions (e.g., PV applications), such as the incorporation of carbonised templates into the silica framework. More recently, metal doping, such as cobalt and iron, was introduced to further strengthen the hydro-stability and even enhance the ammonia selective features.

**Figure 2:**
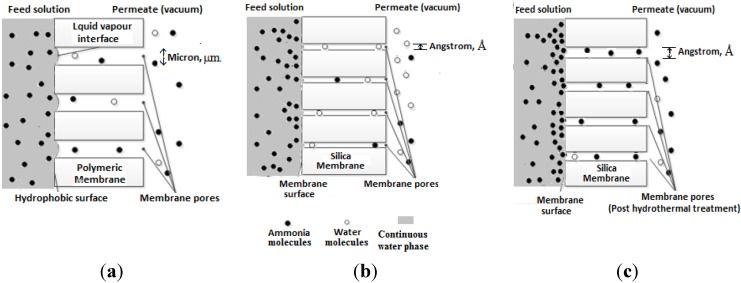
A comparison of ammonia transport mechanisms through membranes: (**a**) the porous hydrophobic membrane (pore size range of micrometres); (**b**) the ammonia rejective molecular sieve silica membranes (pore size range of angstroms); and (**c**) the ammonia passive silica membranes (pore size range of angstroms).

A comparison of the separation mechanisms of an ammonia-water system is illustrated in Figure 2, through conventional porous hydrophobic membranes (Figure 2a), dense (ammonia rejective) molecular sieve silica membranes (Figure 2b) and wide pore (ammonia passive) molecular sieve silica membranes (Figure 2c).

## 4. Results and Discussion

### 4.1. Membrane Characterization

Table 1 gives the basic features for the membranes described in Section 2.1, including the fibre/tubular dimensions, surface contact angle, porosity and pore size (for polymeric membrane) or gas separation factor (for silica-based ceramic membrane). It can be seen that the original silica-based ceramic membranes, CoSi and FeSi, have He/N_2_ permselectivities of three and five, respectively. As these exceed the Knüdsen value of 2.6, this indicates that the membranes possess a molecular sieving structure capable of separating He (molecular size of 0.26 nm) from N_2_ (molecular size of 0.364 nm). Hence, it is anticipated that these two silica membranes will exhibit a higher selectivity towards water molecules (a similar kinetic diameter to He). The PP membrane has a contact angle of 118° ± 6°, indicating its high hydrophobicity, which is advantageous for preventing water intrusion into membrane pores (*i.e*., pore wetting).

### 4.2. Performance of Original Silica-based Membranes (with Synthetic Solution)

#### 4.2.1. Permeation Flux *versus* Feed Temperature

**Figure 3:**
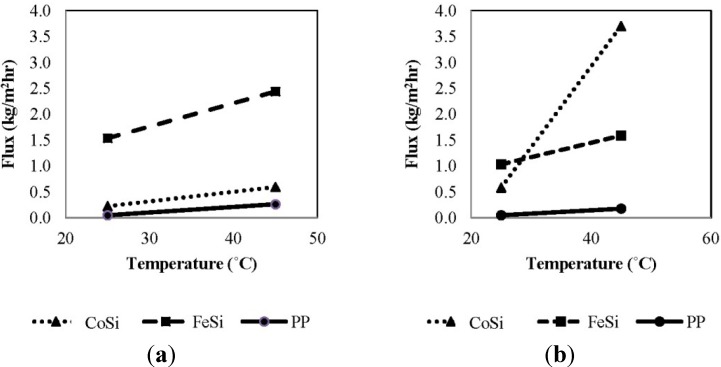
The effect of feed temperature on the total permeation flux of various membranes using synthetic feed solutions (permeate absolute pressure 100–150 Pa): (**a**) 50 mg/L ammonia; and (**b**) 1000 mg/L ammonia.

Figure 3 shows the effect of feed temperature on the overall permeation flux for various membranes using synthetic aqueous solutions as the feed. In general, the total permeation flux (ammonia + water vapour) increases with increasing temperature from 25 °C to 45 °C, regardless of the membrane type and ammonia concentration. In Figure 3a, with a 50 mg/L ammonia concentration, the FeSi membrane shows the highest permeation flux at the same operating temperatures of 25 °C (ambient temperature) and 45 °C, which is approximately 10-fold higher than that of CoSi and the benchmark polymeric membrane. This may be associated with the higher He/N_2_ gas separation factor of the FeSi and, hence, a higher selectivity towards water vapour (Table 1) and less competitive adsorption from ammonia; while for the PP membranes, the low flux is perhaps due to the unoptimized operating conditions and the poor module design in which better performance is expected in VMD when membranes and flow conditions are optimal [35,36]. Similarly, Figure 3b shows that FeSi membrane has the highest flux at 25 °C with an ammonia concentration of 1000 mg/L. Surprisingly, the CoSi membrane shows an unusual 7.5-fold flux increase from 25 °C to 45 °C, which is about 2.5-fold higher than that of FeSi. This may be due to the membrane material degradation affecting the silica pore structure and, subsequently, the separation function. Nevertheless, further investigations are needed to confirm this phenomenon.

Overall, the permeation flux for the FeSi and PP membranes decreases with increasing ammonia concentration; the main reason being that for these membranes, which were claimed to be water-selective, the vapour flux is strongly affected by the water content in the solution. Although ammonia has a relatively lower possibility of entering the membrane pores, it still may act to inhibit water flux at the pore surface.

#### 4.2.2. Water/Ammonia Selectivity of Various Membranes

**Figure 4:**
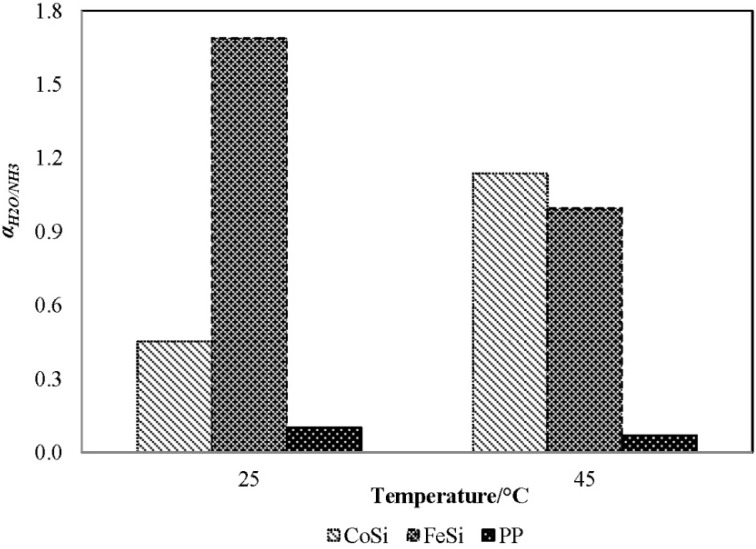
The water/ammonia separation factor, α_H2O/NH3_, of the CoSi and FeSi membranes at different feed temperature (synthetic solution of 1000 mg/L ammonia concentration; permeate absolute pressure, 100–150 Pa).

Figure 4 presents the water/ammonia separation factor [Equation (5)] for various membranes with a feed solution of 1000 mg/L ammonia at various feed temperatures. The permeate compositions were obtained by analysing the permeate results presented in Figure 3. Clearly, the FeSi membrane shows the highest selectivity towards water [*i.e*., α_H2O/NH3_ of 1.7, Equation (5)], which is consistent with its higher gas selectivity presented (Table 1). On the contrary, the benchmark PP membrane shows the lowest water/ammonia separation factor (<1/10), corresponding to its hydrophobic feature and, thus, operating according to the distillation separation (ammonia selectivity over water), as water has a higher boiling point than ammonia (100 °C for water *versus* −33.4 °C for ammonia at atmospheric pressure).

In general, the water selectivity decreases with increasing temperature. This is because at a higher temperature, the ammonia is less soluble in water under the same partial pressure, which favours the release of free ammonia, and hence, the selectivity toward water decreases [Equation (3)]. Interestingly, the CoSi membrane shows the opposite trend: the water selectivity increases rapidly from 0.45 to 1.16 with increasing temperature. Again, the results agree well with the experimental observation in Figure 3b, in which a sudden 7.5-fold flux increase indicates a possible degradation of the silica pore structure and, hence, a major loss in ammonia selectivity.

### 4.3. Performance of the Hydrothermally Treated Silica Membrane

#### 4.3.1. Pure Water Flux

**Figure 5:**
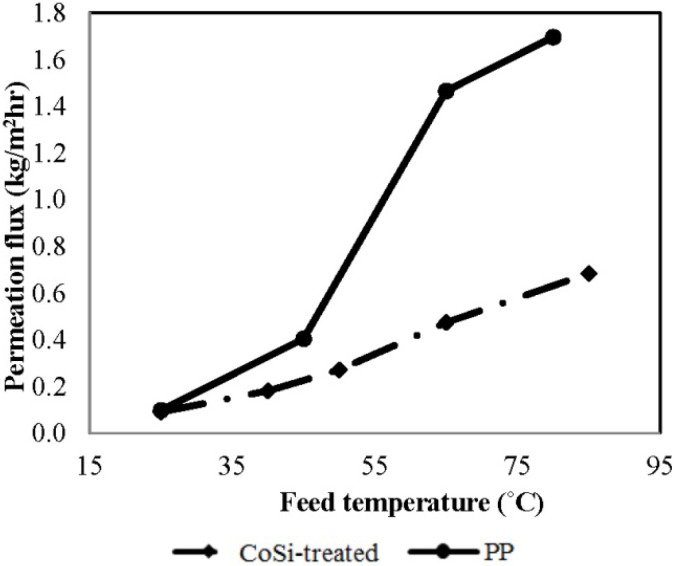
The effect of feed temperature on pure water flux for PP and CoSi-treated membranes [feed, deionized (DI) water; permeate absolute pressure, 100–150 Pa].

Based on the previous results presented in Figure 3 and Figure 4, both silica membranes showed ammonia blocking features, due to the molecular sieving function of the silica structure. While this function would be useful to instead capture water and keep ammonia on the feed side, the pore size range is not suitable for another desired feature, to permeate concentrated ammonia. As previously mentioned in Section 2.2, by slightly widening the pore size to a range of 0.3–0.5 nm using hydrothermal treatment in 90 °C liquid water for several hours, it is possible to overcome the size exclusion mechanism and achieve a competitive ammonia adsorption to efficiently block the permeation of water vapour. To exclude the influence of ammonia transport, DI water was used as the feed to investigate the effect of feed temperature on the pure water flux for the hydrothermally-treated silica membrane, namely CoSi-treated, and the benchmark PP membrane, as illustrated in Figure 5. Similar to the performance of any other MD membranes, a classic increasing trend of permeation flux with increasing temperature is observed for both membranes. Interestingly, although the same initial flux at a low temperature of 25 °C is observed, the benchmark PP membrane achieved a 2.5-fold higher flux than the silica membrane at 80 °C. Differences are likely to be due to the very different mechanisms, where the PP membrane was working according to free surface distillation, the CoSi-treated membrane was working according to molecular diffusion.

#### 4.3.2. Concentration Factor *versus* Feed Temperature and Operation Time

**Figure 6:**
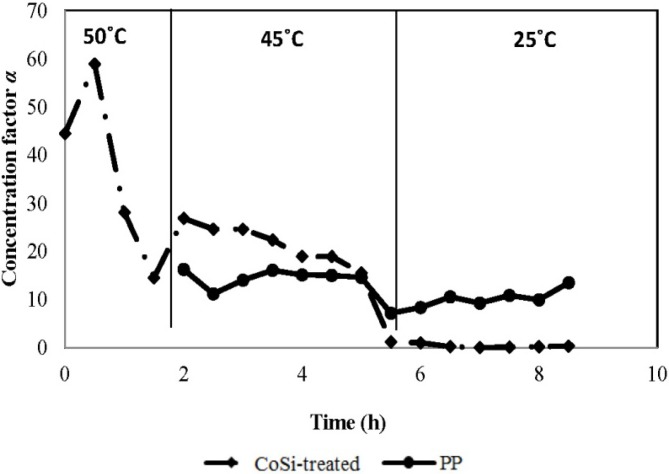
Concentration factors α of CoSi-treated and PP membranes along operation time (synthetic feed solution, 1000 mg/L ammonia; permeate absolute pressure, 100–150 Pa).

To examine the ammonia selectivity of the CoSi-treated membrane, experiments using 1000 mg/L of synthetic feed solution were conducted at varied feed temperatures in a descending sequence of 50 °C, 45 °C and 25 °C. With the PP membrane as the benchmark, the performance results in terms of the concentration factor of the downstream product along operation time are analysed and given in Figure 6.

The CoSi-treated membrane exhibited extraordinary selectivity towards ammonia in the beginning of the test at a feed temperature of 50 °C (initially increasing from 44-fold to 60-fold, corresponding to 50,000 mg/L or 5 wt % ammonia concentration in the permeate). Although a dramatic decrease is observed in the first 1.5 h of operation, the CoSi-treated membrane still shows an encouraging averaged concentration factor of 37-fold and has reached a reusable ammonia concentration level. This is mainly attributed to the combined transport mechanism of molecular diffusion and interactive ammonia-silica adsorption. As the operation continues at a slightly lower feed temperature of 45 °C, relatively stable performance is observed and an averaged 22-fold concentration is achieved, corresponding to 18,000 mg/L of ammonia concentration, which is more than 23 times higher than that of the untreated CoSi membrane (Figure 4) and 50% higher than the benchmark PP membrane. However, for the CoSi-treated membrane, a continuous operation at room temperature at 25 °C shows no preference towards ammonia.

Generally, the concentration factor for ammonia in the downstream product increases with increasing temperature for both membranes, but decreases with the operation time, especially for the silica membrane. The PP membrane shows fairly stable separation performance with steady temperature variation, following a distillation mechanism. There are two possible reasons for the changing ammonia separation results of the silica membrane over time: (1) at ambient temperature, the ammonia adsorption to the silica sites is more predominant than desorption, and thus, ammonia might be trapped in the pores; (2) potential degradation of material/pore structure of the silica matrix occurred after several hours of operation, due to overexposure to the hydrothermal conditions that caused unnecessary pore widening. Nevertheless, further surface inspection is needed to confirm the above hypotheses.

## 5. Conclusions

With the ultimate goal of capturing free ammonia from industrial wastewater to a reusable concentration level, this preliminary study has explored the potential of concentrating ammonia using molecular sieving membranes in PV mode.

It was found that the iron doped silica membrane obtained the highest permeation flux; while the benchmark PP membrane had the lowest. Further analysis on the permeate concentration suggested that the FeSi has exhibited the highest water/ammonia selectivity (*i.e*., lowest ammonia selectivity) while the PP membrane presented showed the highest ammonia selectivity. This was consistent with the gas separation features of the two studied molecular sieve silica membranes with a pore size smaller than that of the molecular size of ammonia (0.326 nm).

After undergoing a hydrothermal treatment, the CoSi-treated membrane had demonstrated an extraordinary selectivity towards ammonia, *i.e.*, up to a 60-fold higher concentration in the downstream product, which has exceeded that expected from free surface distillation. A highly concentrated solution with 5.0 wt % of ammonia could be achieved for potential reuse from a feed solution containing less than 1000 mg/L of ammonia. However, the long-term performance of the CoSi-treated membrane showed signs of potential material/structure degradation, which might be associated with overexposure at hydrothermal conditions, especially at a relatively high temperature. Although the hydrophobic polymeric membrane showed a fairly stable separation performance (an ammonia concentration factor of 14-fold), it cannot exceed distillation limits, due to its large pore size and, thus, no influence at the molecular level.

Overall, PV has a great potential to effectively remove ammonia from aqueous solution. The incorporation of molecular sieve silica-based ceramic membranes has greatly enhanced the ammonia permeating through the membrane for concentration downstream. Yet, further efforts are required for improving the long-term performance and material stability to accomplish a highly efficient single-step ammonia capture process, such as: (1) the high ammonia stability of the membrane structure; (2) new strategies on precisely controlling the pore size to a desired range; (3) establishing a mathematical model for ammonia transport through the silica matrix; and (4) the development of advanced membrane characterization techniques for identifying the pore widening effect and the cause of silica structure degradation.

## Nomenclature

*A*: Membrane permeability constant
*B*: Membrane permeability constant
CoSi: Cobalt coated silica membrane
FeSi: Iron coated silica membrane
*MW*: Molecular weight (g/mol)
*N*: Permeation Flux (mol/m2·s)
*P*_avg_: Average pressure in membrane pore (Pa)
*P*_sat_: Saturation pressure (Pa)
PP: Polymer membrane
*P_p_*: Pressure in permeate stream (Pa)
*R*: Universal gas constant (m3·Pa/Kmol)
*T*: Temperature (K)
μ: Viscosity (Pa·s)
